# Locating farmer-based knowledge and vested interests in natural resource management: the interface of ethnopedology, land tenure and gender in soil erosion management in the Manupali watershed, Philippines

**DOI:** 10.1186/1746-4269-3-30

**Published:** 2007-09-05

**Authors:** Lisa Leimar Price

**Affiliations:** 1 Department of Social Sciences, Wageningen University, P.O. Box 8060, 6700 DA Wageningen, The Netherlands

## Abstract

This paper examines local soil knowledge and management in the Manupali watershed in the Philippines.

The study focuses on soil erosion and its control. Research methods used in the study include ethnosemantic elicitations on soils and focus group discussions. In addition, in-depth work was conducted with 48 farmers holding 154 parcels at different elevations/locations in the watershed. The on-parcel research consisted of farmer classifications of the soil, topography, and erosion status of their parcels. Soil samples were also taken and examined. Farming households were also examined with regard to erosion control activities conducted by age and sex. Erosion management was examined in relation to tenure of the parcel, which emerged as a salient aspect among focus group members and was evidenced by the actual control measures taken on farmed parcels.

The results show that the major constraint in soil erosion management is not local knowledge as much as it is the tenure arrangements which allow "temporary owners" (those working rented or mortgaged parcels) to manage the parcels as they see fit. Most of these temporary owners are not willing to invest in erosion control measures other than water diversion ditches. Parcel owners, in contrast, do invest in longer term erosion control measures on the parcels they actually work.

The findings of this paper illustrate that linking local knowledge and practices is often not sufficient in and of itself for addressing questions of sound environmental management. While local knowledge serves farmers generally well, there are some limitations. Importantly, the pressures in the contemporary world of markets and cash can undermine what they know as the right thing to do for the environment.

## Background

Ethnopedology includes the study of soil folk knowledge (cognitive systems or corpus), local soil management (praxis), and beliefs (cosmos) [1-5]. Because ethnopedology falls also under the more encompassing ethnoecology, land management is part of this domain [6]. Importantly, however, actual management practices may be linked to other factors beyond corpus and cosmos, most notably material parameters, but also other cultural and social parameters [1,6,7]. Thus, an assortment of various antecedents and contemporary circumstances (historical, socio-economic, cultural, and environmental) come together and impact both perceptions of the environment and environmental actions [2,8,9].

This paper specifically examines the problem of soil erosion as linked to knowledge and management practices in the Manupali watershed on the island of Mindanao, the Philippines. Some of the questions that guided the research were: 1) how do farmers classify soils, particularly with regard to fertility and erosion; 2) what do they view as the cause of erosion; 3) how do they control erosion; and 4) what are the opportunities and constraints to erosion control, including other socio-cultural and socio-economic factors. The research process began in an inductive manner with only the above questions rather than hypotheses to test. Tentative explanations of observations and preliminary analysis of selected data during the course of the research generated several hypotheses for further investigation and testing.

The research reported on in this paper was conducted under the umbrella of the Sustainable Agriculture and Natural Resources Management Collaborative Research Support Program (SANREM CRSP) started in 1991 and currently encompassing 11 countries through 2009 [10].

### Research area

**Figure 1:**
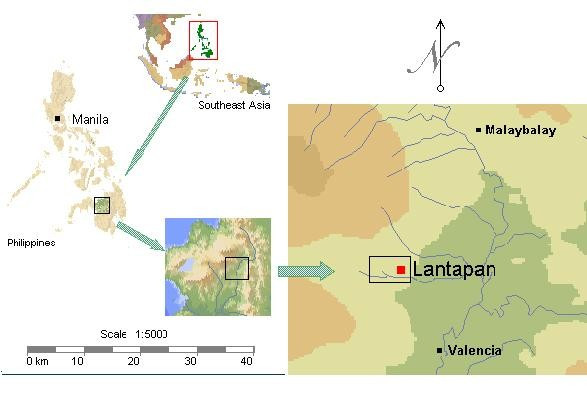
**The Philippines, island of Mindanao and Lantapan**. Source: SANREM CRSP Southeast Asia picture archive. Retrieved July 20, 2007 from

**Figure 2:**
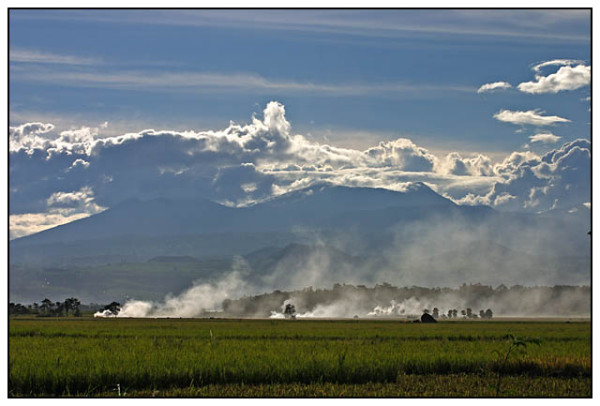
**Mt. Kitanglad, Bukidnon**. Used with permission of B. Timonera, photographer.

**Figure 3:**
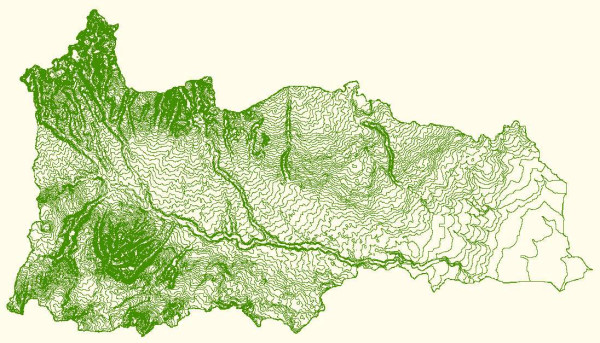
**Contour map of the Manupali watershed**. Source: SANREM CRSP Southeast Asia picture archive. Retrieved 9 February, 2007 from

The Manupali watershed is located in the municipality of Lantapan, Bukidnon Province on the island of Mindanao in the Philippines (Figure 1). The watershed comprises 31,820 hectares and covers the entire municipality (44% of the watershed area) [11,12]. The area consists of multiple elevations ranging from river flats, up through a mid section of rolling hills, to mountain sides in a high mountain range stretching to the peak of Mt. Kitanglad (Figure 2). Roughly 40% of the land is steeply sloping (40+ %) and 30% is rolling to hilly (Figure 3).

Starting from the upper elevation of the watershed, the area consists of steep slopes with some moderately rolling areas (1600-nearly 3000 m) comprised of primary forests. The upper extent consists of the Mt Kitanglad Range National Park. There is (encroaching) potato production at the lower forest margins. Below this in elevation is secondary growth at the forest margins with moderately rolling to steeply sloping topography. At this elevation of 1300–1600 meters, corn and vegetable production predominate. Next, at an elevation of 950 to 1500 meters, are rolling grasslands which are used for intensive maize cultivation. At an elevation of 400 – 500 meters the topography encompasses more gentle rolling hills with some areas that are moderately steep. This area is used for a variety of crops including maize. Lastly, there is the lowland area of the watershed which is flat with some minor gentle rolling landscape where the Manupali River discharges into the Pulangi River. The lower land areas are serviced by the Manupali River Irrigation System [12]. Rice and sugarcane are grown in the lower areas. A small amount of coffee is also grown at various elevations.

The trend in land use has been an expansion of agricultural land and a reduction in forest in the watershed. In 1973 forests made up 51.4% of the watershed and agriculture 28.4%, but dense forest made up 29.3% in 1994 and agriculture 49.7% of the landscape [13]. Further, the trend also shows an increase in cultivation on steeper slopes. The consequence of these trends is thought to be an increase in the likelihood of severe soil erosion and sedimentation downstream [12,13].

The study area population is comprised of the indigenous population (Talaandig, also known as speakers of Higaonon). The Talaandig reside in the upper elevations in municipalities surrounding Mt. Kitanglad and Lantapan in one of these municipalities [14]. A host of in-migrants, arriving first from the island of Luzon in the 1950's and then from the Visayan Islands (Cebu Bohol and Leyte) by the 1970's, reside and farm in the watershed. Most of the in-migrants were from depressed rural areas seeking land or employment and generally a better life [15].

The municipality of Lantapan has 14 barangays, which is the smallest government unit in the Philippines. Each barangay has an elected official called a Barangay Captain. A barangay has area sub-divisions that are called Puroks. Each Barangay Captain keeps official records on his community.

## Methods

There were a number of primary research activities implemented: 1) locating farmers engaged in agricultural activities at multiple elevations throughout the watershed and interviewing a representative sample through topic guideline interviews; 2) constructing farmer discussion groups with the aid of the farmers themselves via sorting through numerous variables to ensure that group members had similar vested interests in the areas of farming and conservation, including gender and ethnicity; 3) conducting focus discussion group sessions with the various vested interest groups on issues of soil and water management, including constraints and incentives to the viable management of soil degradation; 4) interviewing key informants on the classification of soils and the farming environment; 5) collecting data from household members across the landscape and cropping systems about their farms and soil management activities disaggregated by gender and adult/child; 6) matching of indigenous soil classification with the history of farmer's parcels; 7) matching of indigenous soil classification with soil sample analysis.

Farmers engaged in activities at multiple levels in the watershed were located and a representative sample was interviewed using topical guidelines. Barangay officials were interviewed as key informants in the 14 barangays of Lantapan. The officials gave the names of 259 multiple parcel farmers farming 593 parcels, which were classified into: farmers farming at different locations/different crops (n = 45); farmers farming at different locations/same crop (n = 65); and farmers farming at same location/different crops (n = 28). Same location indicates that farmers are farming in the same puroks, while those farming in the same barangay are at different locations/elevations as indicated by the informants.

A sample of 48 farm households was selected out of the 259 possible (above). The farmers were selected because they were representative as farming multiple parcels throughout various locations in the landscape. There were a total of 154 parcels being farmed by the 48 farmer households, indicating that the sample farmers have an average of more than 3 parcels per farm household.

Six focus discussion groups were formed according to different criteria relative to the information in individual interviews. Group I is composed of male and female farmers who are holders of multiple parcels and are engaged in renting/leasing parcels from others (standard term in the watershed being 3 years). Their farming activities are market production/profit orientated rather than subsistence orientated. In addition, none of their spouses are engaged in farm field cultivation. Two households are engaged in farming throughout various barangays in the landscape. They all belong to the Visayan culture/language groups.

Group II members farm only the land they own and hire labor. Members of this focus group manage the farm on their own or depend on adult sons (who have their own farms) to manage the farms or to aid on the farm when the son is clear of his own obligations. Thus, they utilize hired labor and they have family management. Two farm households have small grocery stores and all indicate self-financing. In addition, one farm household finances several small farmers growing vegetables. Members of this group are all Talaandig men.

Group III is composed of female household heads who are actively engaged in farming with their husbands. They are all Cebuana. Group IV is a group of male rice farmers in the irrigated area. All have rented farms, but two of the group members also own parcels of farm land at other locations in the watershed that they rent to others. All farmers hire labor.

Group V are all male Talaandig owner operators planting coffee and maize. Group VI are Talaandig women planting coffee and maize except one woman, who grows vegetables for market sale. All farm their own parcels of land with mostly family labor and family management.

Key informant interviews were utilized to collect soil classification/characterizations with 28 individuals in their homes. This was followed by on-parcel identification of soils according to folk characterizations conducted with a sample of 40 (randomly selected) farmers on their primary parcel(s) (43 parcels). Farmers were asked to describe their parcel according to soil color, topography, soil texture and fertility. Additionally, soil samples were taken from each parcel in the presence of the farmer and color matched to the Munsell soil color charts by farmers. The parcel was then geo-positioned and the soil samples were sent to a specialized soil laboratory for analysis. The on-parcel research covered the various landscape agro-ecological zones/landscape positions in the watershed (number of parcels = 8 forest margins, 11 cropped grasslands, 19 with a permanent area of maize and sugarcane cultivation, and 5 maize and rice croplands. Two parcels had coffee at the time of the interview).

Selected tests were conducted on data from the 43 parcels. Correlations were conducted between the outcome of the laboratory analysis of soils (pH and nutrients) and farmers color classification of the soil sample. Spearman rank correlations were performed on the variables of: 1) slope and erosion and 2) parcel fertility and erosion severity. The Chi Square Goodness of Fit test was performed on erosion control measure by tenure status of the parcel.

## Results

### Farmer's perceptions and the causes of soil erosion

Farmers in their individual interviews identified the causes of soil erosion (Table 1). Factors identified as important included rolling and sloping topography and continuous and heavy rains. These two were the most popular responses. Causes directly linked with farmer's agricultural practices included continuous cultivation, use of tractors, and deforestation.

**Table 1:** Farmer's perceptions of causes of soil erosion. N = 48 farmers.

Causes	Percent reporting
Rolling/sloping topography	30.23
Continuous/heavy rain	18.60
Continuous cultivation	18.60
Use of tractors	13.95
Deforestation	11.62
Hard/compact soil	2.30
Lack of run-off canal	2.30
Farmer laziness	2.30

Based on farmer ranking of the erosion severity of their parcels and each parcel's slope, a Spearman rank correlation illustrates the consistency in their perceptions with the greater slope indicating greater erosion, and is statistically significant (at .001). Likewise, their ranked perceptions of parcel fertility and erosion severity show statistical significance; the greater the fertility the less the erosion (sig. at .001). These correlations illustrate consistency in farmer's perceptions and statements regarding fertility, erosion, and topography.

### Folk soil classifications, parcel soil samples, and data correlation

Twenty-eight farmers were interviewed on folk soil classification. These interviews followed ethnosemantic elicitation procedures common in anthropology. No hierarchical taxonomy is evident and the organization is more along paradigmatic lines. Two soil types consistently emerged for the three ethnic groups of Talaandig, Cebuano, and Boholano, with a primary dichotomy of good and bad soils with distinctions in color (black for good/fertile and red for bad/infertile). Soil qualities of texture and moisture and plant growth (vigour, size, leaf color) were used as soil fertility indicators (Additional file 1: Local soil classification).

Soil acidity of the 43 soil samples from the on-parcel work had a pH range from 4.5 to pH 5.9. The mean pH in different landscape positions were pH 5.1 in the forest margin, pH 5.2 in the grassland, pH 5.0 in the corn area, and pH 5.2 in the in the paddy area. Results on soil chemical properties showed that the forest margins and grassland have slightly higher organic carbon (OC) contents (2.32% and 1.98%, respectively) than the corn (1.66%) and paddy area (1.64%). Across farms, the mean available nutrients were nitrogen (0.19%), phosphorous (10.39%) and potassium (0.66%).

Farmer soil classifications on their parcels as red or black did not correlate significantly with the laboratory soil analysis. This may be in part due to the fact that the soil horizon change is quite subtle and farmers are not immediately aware of the loss of topsoil. The color range, however, does move from black to red as one moves down from the topsoil into the subsurface [16,17].

Folk soil classification of soil texture on their parcels, on the other hand, shows areas of overlap with the scientific classification of soil texture. Sticky is a quality of both infertile and fertile soils according to farmers. This is understandable given the nature of the prevailing soil in Lantapan. Adtuyon clay is sticky and covers the project area from the lower elevations up to Cawayan. Kibangay and Basak are Kidapawan clay loam and are also sticky [16]. In addition, a statistically significant positive correlation was in evidence for farmers' classification of their parcel topography (flat, flat to slightly rolling, rolling, etc.) and soil pH (significant at .01).

### Soil erosion control activities

**Figure 4:**
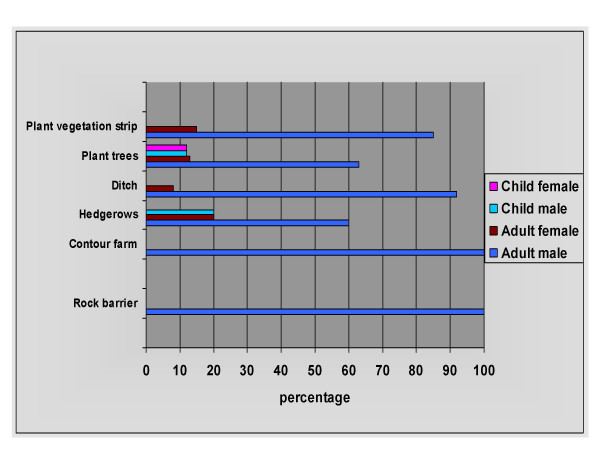
**Erosion control measures by sex and age**. N = 96 (48 men and 48 women as farming couples and their children).

From the sample of 48 farmers, men listed their actual contribution to soil erosion control measures ranging from 60 to 100 percent responsibility for the measure and they engaged in all listed activities. Women listed tree planting (13%), planting vegetation (15%), constructing ditches (8%), and hedgerows (20%). Children engaged in some of the measures conducted by mothers including tree planting, with boys at 12% and girls and 12%, and hedgerows with boys at 20% (Figure 4).

### Tenure and soil erosion control measures

**Figure 5:**
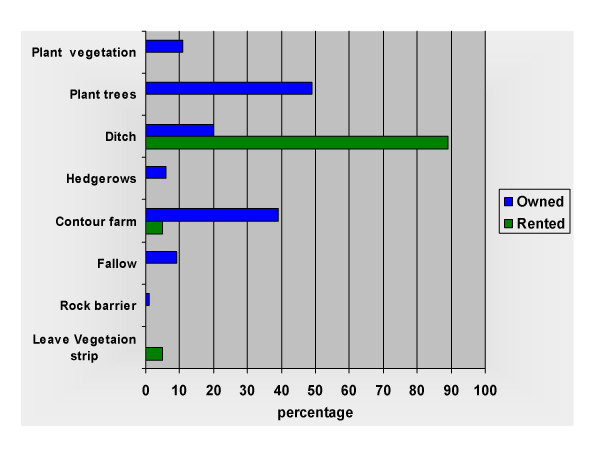
**Erosion control measures by tenure**. N = 48 farmer households.

Tenure status of a farmed parcel has a significant effect on a farmer's willingness to invest in erosion control measures. Among the 48 sample farmers, 22% of their parcels were being rented out and 9% were mortgaged out. Ten percent of the parcels were under the control of sample informants who were renting them. There is a shortage of land for sale and market oriented farmers rent parcels for commercial production, many of whom use tractors. Farmer's are more willing to invest in labor and capital-intensive soil conservation measures on their own land. Erosion control and water control actually engaged in by farmers on rented parcels included a water diversion ditch (89% of rented parcels), contour farming (5%), and leaving a strip of vegetation when ploughing (5%). Those farming land they owned, however, implemented more diverse and longer term strategies. Owners planted trees (49%), contour farmed (39%), installed water diversion ditches (20%), left fields fallow (9%), planted hedgerows (6%), and constructed erosion barriers from rocks or wood (1%) (Figure 5).

The Chi-square goodness of fit test was used to examine the difference between the observed and expected number of observations that fall into each category of erosion control measure by tenure status. The hypothesis that responses on type of control measure utilized will differ in frequency based on tenure status is verified (statistically significant at the .001 level).

Farmers unanimously reported that renters would not invest in more costly and longer-term soil erosion control measures. Commercial farmers felt that they had no incentive to protect the soil they rented from other farmers for commercial production. Even contour ploughing was not considered because they said they preferred to use tractors and that contour farming takes time. Commercial farmers also said that hedgerows took up parcel space that could be used for production, and tree planting was expensive (cost of trees). What emerged in the focus groups was that current rental (or mortgage) agreements allow the renter (or "temporary owner") to do whatever they like to the parcel, including removal of hedgerows and trees. Most who rent out (or mortgage) parcels use the money to purchase inputs for their other parcels, but the tight land market would indicate that owners of the parcels could have some leverage.

Tractors are thought to contribute to soil erosion. Tractors are not only a preferred means of ploughing, but one that also holds high social status. The ability to afford to own or rent a tractor is an indication of wealth and thus social standing. Most tractor use occurs among wealthier farmers who are market oriented. It is, however, the land they obtain as "temporary owners" for production and not their own that suffers the most.

### The focus groups and perceptions

In addition to tenancy as a disincentive to soil conservation, scarcity of capital and relative return for labor emerged as constraints in the discussion groups. Capital was cited as a constraint to soil management by farm land owners and, more frequently, by renters/mortgagees. Some measures farmers thought of to ease the capital problem were: 1) a government sponsored tree planting program coupled with an information drive to educate farmers on conservation; and 2) establishment of credit options to ease farmers' financial constraints, but the rate of interest must not be very high.

Relative return to labor was an issue among poorer subsistence oriented Talaandig farmers (Group V). They felt that seeking off-farm employment for additional income was a priority over and above investing time and money in soil management. The group felt that an important incentive for poorer farmers to invest in soil management would be for the government to give them seedlings for free with assurance that they the farmers would have sole ownership of the trees. Multipurpose trees were important to the group. They are willing to plant fruit-bearing trees like mango and coffee so they can earn income while the trees grow. Trees should also provide a source of fuel for cooking. The group believes that this suggestion is viable, provided that labor as well as capital investment issues are addressed.

### Gender and age in soil conservation

Talaandig women, on the other hand (Group VI), thought that the use-value of trees was incentive enough for landowners to plant them. They felt, however, that they did not know enough about conservation farming and that outreach education was needed. Their opinions on owner/renter profit sharing of tree crops at 50:50 was the same as all the other groups, but in addition, they thought that extending the term of contract by one additional cropping in conjunction to profit sharing of tree produce would be more attractive.

Education came up as an issue among other women. The discussion group of Cebuana women farmers (Group III) said they had heard of contour farming but did not know how to do it. Most extension courses are filled with male farmers even though women attend from time to time. They thought that courses for women only on the same conservation farming subjects taught to the men would be more comfortable and desirable. The current conservation measures practiced by the women include constructing water run-off earthen ditches and planting bananas in ditches. While one member planted trees around her parcel boundaries, the women noted that trees are expensive to obtain.

## Discussion and Conclusion

The results of this study with farm families of the Manupali watershed reveal some important aspects for understanding knowledge and behaviour in soil erosion management.

The elicited characteristics of soils (with regard to fertility) show that all three ethnic groups had similar categories of characteristics. Soil color and soil texture that are used to indicate fertile and infertile land feature prominently (most salient). Plant health also features prominently as an indicator of soil fertility in association with soil color and texture. This case does not appear unusual, however, as both color and texture emerge as the most salient features of soil classification across cultures [1,2]. The use of vegetative features in the classification of soils evidenced in this paper is also noted as occurring in other contexts [2]. Finally, the classification of soils by farmers in this study as non-hierarchal has been encountered in other contexts [2].

It has been asserted that farmer classifications reflect utilitarian values, such as is proposed for the property of soil fertility by Talawar and Rhoades [2] which this study in general supports. Naming in relation to utilitarian cultural importance has been shown for other subsistence agricultural resources such as crop varieties as well as agricultural pests [18,19]. Soil fertility, however, is a quality that emerges as part of "good" or "bad" soils based on a combination of classificatory aspects. Important also is the farmer's conceptual linking of parcel slope with erosion severity which was statistically significant. Likewise, the rank correlation of their perceptions of parcel fertility and erosion severity show statistical significance; the greater the fertility the less the erosion.

This case study provides evidence that the farmers, across ethnic groups, have some of the observation skills and knowledge of erosion control techniques to aid them in managing soil erosion to a certain extent. This being said, it does appear that some knowledge-based support may be warranted as farmer soil classifications on their parcels as red or black was not significantly correlated with the results of the soil analysis from the laboratory. As noted earlier in this paper, this may partially be due to observation problems farmers have in regard to the subtle color changes in the soil horizon, which makes loss of top soil difficult to detect early on. Women also expressed a need for more knowledge and training in erosion control techniques. It should be noted that women were most engaged in more long-term sustainable measures through tree planting and the planting of other vegetation. The reasons for this, however, were unexplored and thus remain unclear.

The biggest constraints to soil erosion control in the watershed appear to be more socio-cultural and socio-economic. There was a statistically significant positive relationship between erosion control measures in relation to the kind of tenure a parcel was under. In the case of the Manupali watershed, it became clear that multiple parcel holders rent out (or mortgage out) one or more of their parcels to farmers who use them to grow for the market (commercial farmers). The agreements for the rental or mortgage culturally make the farmer a "temporary owner". Under this status, the "temporary owner" has the right to remove vegetation and, according to focus group informants, this includes trees if the "temporary owner" chooses to do so. The agreements on rights of "temporary owners" are socio-cultural. According to focus group discussants, there are additional factors with regard to renting out/mortgaging parcels in relation to soil erosion control. This first is that farmers rent/mortgage out parcels in order to earn funds to invest in the parcels they own and are actually actively farming. There is thus a need for cash as well as access to reasonable farm credit. An economist who reports on his soil conservation research in the Manupali watershed notes that "...credit emerges as an important factor conditioning investments in natural capital...", but "...incentives for soil conservation investments are generally stronger on small farms than large" [[20], p. 275–276].

It should be noted that the farmers who obtain parcels through rental or mortgage for commercial production not only destroy vegetation, but do not engage in planting any. In fact, their primary erosion control method is making water diversion ditches in order to redirect the water flow so their standing crops are not damaged. Most of these farmers also use tractors in land preparation that make the soil hard and compact. On the other hand, the disk plough in common use with the tractors pulverizes the soil and makes it more easily washed away. The return on capital and labor investments were important reasons stated by commercial farmers for why they did not invest more in soil erosion control. Such a finding is important in the context where agricultural expansion is unwittingly encouraged on fragile lands (sloping erosion prone lands), despite the good policy intention of enhancing food security (food sufficiency and price stability), particularly in maize with regard to the Manupali watershed. More land is brought under commercial production, fallows shortened or eliminated, and vegetation cover removed. National "food policies based on price and trade restrictions may also accelerate land degradation by promoting expansion of relatively erosive grain crops" [[21], p.111].

The findings of this paper ultimately illustrate that linking local knowledge (corpus) and practices (praxis) is often not sufficient in and of itself to address questions of sound environmental management. While local knowledge serves farmers generally well to a degree, the pressures in the contemporary world of markets and cash can undermine what they know as the right thing to do for the environment.

## Competing interests

The author(s) declare that they have no competing interests.

## Supplementary Material

Additional file 1 Local soil classifications. Table showing the results of elicitations on soil classifications in relation to soil fertility for three ethnic groups in the Manupali watershed. Click here for file

## References

[B1] Barrera-Bassols N, Zinck JA (2003). Ethnopedology: a worldwide view on the soil knowledge of local people. Geoderma.

[B2] Talawar S, Rhoades RE (1998). Scientific and local classification and management of soils. Agriculture and Human Values.

[B3] Williams BJ, Ortiz-Solorio CA (1981). Middle American folk soil taxonomy. Annals of the Association of American Geographers.

[B4] Toledo VM (1992). What is ethnoecology? Origins, scope and implications of a rising discipline. Ethnoecológica.

[B5] Toledo VM, Steppe JR, Wyndham FS, Zarger R (2002). Ethnoecology: a conceptual framework for the study of indigenous knowledge on nature. Ethnobiology and biocultural diversity: proceedings of the seventh international congress of ethnobiology.

[B6] WinklerPrins AMGA, Barrera-Bassols N (2004). Latin American ethnopedology: a vision of its past, present, and future. Agriculture and Human Values.

[B7] Sillitoe P (1998). Knowing the land: soil and land resource evaluation and indigenous knowledge. Soil Use and Management.

[B8] Brosius P, Lovelace G, Martin G, Martin G (1986). Ethnoecology: an approach to understanding traditional agricultural knowledge. Traditional agricultural in Southeast Asia.

[B9] Price LL (2001). Demystifying farmers' entomological and pest management knowledge: a methodology for assessing the impacts on knowledge from IPM-FFS and NES interventions. Agriculture and Human Values.

[B10] A Brief history of SANREM CRSP. Website managed by Office of International Research, Development and Education, Virginia Tech, USA. http://www.oired.vt.edu/sanremcrsp/menu_aboutus/a%20brief%20history%20of %20SANREM.php.

[B11] Lantapan NRMC (1998). Natural Resources Management and Development Plan (NRMDP).

[B12] Lantican MA, Guerra LC, Bhuiyan SI (2003). Impacts of soil erosion in the upper Manupali watershed on irrigated lowlands in the Philippines. Paddy Water Environment.

[B13] Li B (1994). The Impact Assessment of Land Use Change in the Watershed Area Using Remote Sensing and GIS: A Case Study of Manupali Watershed, the Philippines. Unpublished Masters' thesis.

[B14] Saway V The Talaandig.

[B15] Paunlagui MM, Suminguit V, Coxhead I, Buenavista G (2001). Demographic development of Lantapan. Seeking sustainability: challenges of agricultural development and environmental management in a Philippine watershed.

[B16] Mariano J, Yniguez T, Aguas E (1955). Soil Survey of Bukidnon Province, Philippines Soil Report 21.

[B17] FORI (1982). Hydrological and physical characterization of Muleta-Manupali watershed (Bukidnon). Terminal Report.

[B18] Boster J (1985). Selection for perceptual distinctiveness: evidence from Aguaruna cultivars. Economic Botany.

[B19] Price LL, Björnsen Gurung A (2006). Describing and measuring ethno-entomological knowledge of rice pests: tradition and change among Asian rice farmers. Environment, Development and Sustainability.

[B20] Shively GE (2001). Poverty, consumption risk, and soil conservation. Journal of Development Economics.

[B21] Coxhead I (2000). Consequences of food security strategy for economic welfare, income distribution and land degradation: the Philippine case. World Development.

