# Supplementary material for: Locating farmer-based knowledge and vested interests in natural resource management: the interface of ethnopedology, land tenure and gender in soil erosion management in the Manupali watershed, Philippines
Source: J Ethnobiol Ethnomed. 2007 Sep 5;3:30. doi: 10.1186/1746-4269-3-30 (PMC2034377; doi:10.1186/1746-4269-3-30)
Supplement: Additional file 1 — Local soil classifications. Table showing the results of elicitations on soil classifications in relation to soil fertility for three ethnic groups in the Manupali watershed. [file 1746-4269-3-30-S1.doc]

**Local Soil Classifications**

Talaandig Soil Classification, Lantapan.

Informants: male 8, female 7. Age Range: 39-66 years old

| Fertility | Color | Texture | Moisture | Plant |
| --- | --- | --- | --- | --- |
| Fertile  A  Malambo  Maayad  Madagwag  Lugay na  agkogango  Pualas | A1  Maitum (Black) | A2  Bagalon / Mabagal /  Sibolon (Coarse)  Mahumol (Soft)  Makumo (Form into clod)  Malagkot (Sticky)  Maragway / Masabwag/ Sawaga/Mabunghag  (Loose) | A3  Agtibolon (Form into clod)  Mahamos (Always wet) | A4  Aragi /Maayad puno‑an (Good stand)  Malambo ha sagbot  (Vigorous plants)  Aragiso kayo (Big trees)  Malabo ha pa mola hon (Good crop) |
| Infertile  B  Balising‑on  Magasa  Kuminanggil  Mamenos sa klase  Kamaniyan  Damilag  Maraot  Laos | B1  Amen maliga  (Red)  Bulaw (Brown)  Madalag  (Faded red  color)  Maliga‑on (Red) | B2  Alyabok (Dry/fine as dust)  Bungkad / Masawod  (Loose)  Madason sa dralom  (Bottom soil is hard)  Malagkot / Mapilit (Sticky)  Matibolon (Form into clod)  Mayuntok / Nalupot /  Pino (Fine) | B3  Kabungkad  (Dry)  Mahamos‑hamos  (Wet)  Mamara  (Dry) | B4  Atiu‑ay ha pa mola hon  (Stunted crop)  Hari‑on agkabaloy sa  pamulahon  (Plant does not grow well)  Magasa ha pamulahon daw  mga sagbot (Stunted plant growth and  weeds)  Makalawag ha pamulahon  (Plant is yellow in color)  Maraot sa pamulahon  (Stunted growth)  Tagkalawag ha pamulahon  (Yellowish color of the plant)  Magasa ha sagbot  (Thin plants)  Magasa ha kayo  (Thin trees) |

Boholano Soil Classification.

Informants: male 2, female 2. Age Range:52-68 years old

| Fertility | Color | Texture | Moisture | Plant |
| --- | --- | --- | --- | --- |
| Fertile  A  Tambok  Tabunok | A1  Itom (Black) | A2  Bukagay, pughay  (Loose)  Dahol / Tibook  (Coarse)  Dali mabungcag  (Easily loose)  Humok  (Soft) | A3 | A4  Dagko ang puno‑an (Thick/big base of plant)  Itom ang dahon (Dark green leaves) |
| Infertile  B  Niwang  Omaw | B1  Pula (Red) | B2  Dali ma‑umol  (Easy to form into clod)  Gahi  (Hard)  Pilit  (Sticky)  Pino ang grano  (Fine) | B3  Mala  (Dry) | B4  Dalag ang tanom (Plant is yellow in color)  Gagmay/niwang ang puno‑an (Thin/small plant base)  Luspad ang tanom (Plant is pale)  Madalag ang dahon (Yellowish leaves) |

Cebuano Soil Classification.

Informants: male 3, female 3. Age Range:39-66 years old

| Fertility | Color | Texture | Moisture | Plant |
| --- | --- | --- | --- | --- |
| Fertile  A  Tambok  Alomhon  Tabunok | A1  Itom (Black)  Itom‑itom (Blackish) | A2  Basa‑on (Wet)  Dahol (Coarse)  Humok (Soft)  Pilit (Sticky)  Pughay‑bucagay (Loose)  Tibogol‑tibugol (Cloddy)  Tibook (Whole/cloddy) | A3  Basa‑on (Wet) | A4  Abo‑abohan ang dahon (Dark green or ash gray leaves)  Dagko ang puno‑an (Base of plant is big/robust)  Green ang dahon (Green leaves)  Itom‑itom ang tanom (Greenish plant)  Itomon ang dahon (Dark green leaves)  Lunhaw ang tanom (Dark green/vigorous plant) |
| Infertile  B  Daot  Niwang  Enas  Omal  Omaw  Opaw | B1  Pula  (Red)  Pula‑pula  (Reddish) | B2  Bantok ang ilalom  (Bottom soil is hard)  Bas‑bason (Sandy)  Bucagay (Loose)  Gahi (Hard)  Ma‑umol (Can be formed into clod)  Malahon (Dry)  Pilit (Sticky)  Pino (Fine) | B3  Malahon  (Dry)  Ugahon  (Dry) | B4  Dalag ang tanom (Plant is yellowish)  Gagmay ang puno‑an (Thin/small plant base)  Lalag ang tanom (Plant is yellowish)  Pula ang dahon (Reddish leaves)  Pulahon ang dahon (Reddish leaves) |
